# Stiff Person Syndrome: A Case Report

**DOI:** 10.7759/cureus.99327

**Published:** 2025-12-15

**Authors:** Amelia Dorr, Adrian DiVittorio, Kathleen A Clark, Nirav Pansuriya

**Affiliations:** 1 Internal Medicine, Pulmonology, and Sleep Medicine, Alabama College of Osteopathic Medicine, Dothan, USA; 2 Pulmonology and Critical Care, Mobile Infirmary, Mobile, USA; 3 Family Medicine, Alabama College of Osteopathic Medicine, Dothan, USA; 4 Internal Medicine, Mobile Infirmary, Mobile, USA

**Keywords:** anti-gad antibody, autoimmune, gaba-enhancing drug, idiopathic hypersomnia (ih), neurological autoimmune disorders, neurological disorder

## Abstract

Stiff person syndrome (SPS) is a rare, debilitating, progressive autoimmune disorder affecting the central nervous system (CNS). Patients commonly present with muscle rigidity, gait impairment, and painful muscle spasms triggered by sensory stimuli. The classic form makes up the majority of SPS cases and is associated with anti-glutamic acid decarboxylase (anti-GAD) antibodies. GAD antibodies interfere with gamma-aminobutyric acid (GABA) synthesis, leading to the loss of inhibitory signals and contributing to the over-excitation of motor neurons.

We report the case of a 46-year-old woman who presented with recurrent and worsening episodes of sleep paralysis, daytime fatigue, nocturnal choking, and abnormal muscle movements triggered by lights, noises, and open spaces. Past medical history included anxiety and scoliosis. Physical examination revealed no muscle spasticity, but the patient endorsed difficulty with ambulation. A polysomnography was performed to rule out obstructive sleep apnea, and a multiple sleep latency test (MSLT) was initiated to rule out narcolepsy. The patient was diagnosed with hypersomnia. Sleep paralysis and excessive daytime sleepiness are sleep-related disturbances that may occur secondary to neurological or systemic conditions. While these symptoms are most commonly associated with narcolepsy and sleep apnea, they can also arise from a wide variety of neurological or systemic conditions. Treatment for this patient included 10 mg Adderall daily, frequent ambulation, and appropriate sleep hygiene to improve wakefulness. This case highlights the diagnostic complexity of this disorder, demonstrating the importance of clinical awareness. SPS can mimic other conditions, leading to misdiagnosis. This report aims to highlight the impact of early recognition on the initiation of therapy to minimize the progression of the disease. It stresses the importance of a multidisciplinary approach to treatment for the best clinical outcomes.

## Introduction

Stiff person syndrome (SPS) is a rare immune-mediated disorder of the central nervous system (CNS). It commonly presents with painful muscle spasms predominantly affecting the axial and proximal limbs [1]. The muscle spasms are often triggered by an increased sensitivity to external stimuli [2]. The underlying pathogenesis is understood to involve autoantibodies to various components of inhibitory synapses. This leads to impaired functioning through a low level of gamma-aminobutyric acid (GABA) on pre- and postsynaptic neuronal junctions [3]. GABA functions as an inhibitory neurotransmitter; the impairment of GABA results in hyperexcitability in the motor cortex, manifesting as the impaired relaxation of muscles in patients with SPS. The major classifications of SPS include classic, partial variants, paraneoplastic, and progressive encephalomyelitis with rigidity and myoclonus (PERM) [4]. Definitive diagnosis has proved challenging over the years, with widely accepted criteria coming from Dalakas in 2009 [5].

Classic SPS is the predominant form of SPS, making up 70%-80% of cases. It is most commonly associated with glutamic acid decarboxylase (GAD) antibodies [5,6]. It tends to progress gradually. Spasms and stiffness often begin in the thoracolumbar region and extend to the proximal limbs. Individuals may experience gait disturbances and report walking resembling a “tin-man.” Unexpected tactile, visual, or acoustic stimuli can contribute to exaggerated startle responses. Due to a lack of control over muscle spasms, many patients develop task-related phobias and anticipatory anxiety [1].

The prognosis can vary based on a multitude of factors, including clinical presentation and time of diagnosis. Women are twice as likely to be affected compared to men and are usually affected between the ages of 30 and 40. Comorbid autoimmune diseases are also commonly seen, including diabetes and thyroid dysfunction [7]. The standard of care involves symptomatic treatment with GABA-enhancing drugs or immunotherapy. Depending on the severity and prognosis of the patient, these lines of treatment can be used individually or in conjunction with one another. Benzodiazepines are considered the mainstay of treatment, while baclofen is often used as second-line therapy. Both of these drugs are GABAergic, promoting the relaxation of muscles and reducing spasms. Intravenous immunoglobulin (IVIG) and plasmapheresis are also considered in patients with refractory disease [7].

## Case presentation

A 46-year-old woman presented to the clinic for a follow-up with progressive episodes of sleep paralysis, as well as intermittent episodes of gasping and choking with sleep. Despite the paralysis, she is aware of her surroundings during these episodes. She is unsure of how long each episode is and notes variations of duration during episodes. The episodes have been ongoing for several years; however, the frequency of episodes seems to be increasing. She also endorsed muscle spasticity and tightening. She also experiences fear with returning to sleep and increased anxiety leading up to sleep. Certain lights, noises, and big open spaces are triggers for her muscle symptoms and movements. She also noted excessive daytime sleepiness. At her initial visit three months prior, the patient presented with similar symptoms, except she denied daytime sleepiness at that time. Significant medical history included anxiety and scoliosis. A diagnosis of stiff person syndrome was confirmed by the combination of her symptoms and positive anti-GAD antibody. She was prescribed clonazepam, baclofen, carbamazepine, and IVIG treatments every three weeks. Her BMI was 21.92 kg/m². On examination, the patient did not have muscle rigidity and spasms. Her symptoms often occur in the morning upon waking. Due to worsening symptoms, as well as difficulty maintaining balance, the patient does use a walker to improve ambulation.

Hypersomnia is commonly associated with stiff person syndrome. A sleep study test was ordered to further investigate the causes of the patient’s excessive daytime drowsiness. Prior to the sleep study test, the patient discontinued clonazepam and hydroxyzine due to their ability to induce sedation. While there was some snoring, there was no evidence of sleep apnea. A daytime nap test was conducted to check for narcolepsy, which can induce sleep paralysis. The patient fell asleep quickly during all five nap attempts, averaging eight minutes to fall asleep. However, the patient did not enter rapid eye movement (REM) sleep in at least two naps, thus disqualifying her from a narcolepsy diagnosis. The patient continued to experience sleep paralysis episodes and excessive sleepiness, which were believed to be partially attributable to her stiff person syndrome. The patient was prescribed Adderall 10 mg per day to improve wakefulness and reduce fatigue.

## Discussion

This case highlights the complexity of SPS and its management. The pathogenesis of SPS has been explained by B-cell-mediated autoimmune inflammation that affects different components of inhibitory GABAergic neurons and their synapses. The production of autoantibodies against antigens involved in GABA synthesis and release within the central nervous system results in a dysfunction of major inhibitory pathways. This leads to impaired truncal and axial muscle relaxation due to hyperexcitability. Anti-GAD antibodies are found in 60%-80% of classic SPS cases, attacking the GAD enzyme and ultimately inhibiting the synthesis of GABA. The reduction of GABA leads to a loss of normal inhibitory tone on motor neurons. Transcranial magnetic stimulation (TMS) studies show reduced motor cortex inhibition, consistent with low levels of GABA in the CNS. Electromyography (EMG) shows continuous, involuntary firing of both agonist and antagonist muscles at the same time due to impaired inhibitory signals [2,8]. This loss of inhibitory control causes nervous system hyperexcitability, resulting in exaggerated startle responses and painful spasms triggered by stimuli or stress.

The autoimmune nature of SPS is further supported by its frequent association with other autoimmune disorders, including but not limited to type I diabetes, Hashimoto’s thyroiditis, pernicious anemia, and vitiligo [5,9]. Various factors can trigger symptoms by further depleting GABA and increasing nervous system activity. Anxiety and emotional stress can lower GABA reserves and increase muscle tension. Many patients with SPS experience anxiety, which can create a self-perpetuating cycle. As symptoms can be triggered by sensory stimuli, many patients develop agoraphobia (fear of open spaces) due to the unpredictable nature of spasms in public. Exposure to cold temperatures can also trigger muscle spasms. Fatigue is commonly reported by patients with SPS, although it may be masked by other muscular complaints [10]. One study found that patients with SPS with fatigue showed electrophysiological evidence of presynaptic neuromuscular transmission defect, while patients with SPS without fatigue had no electrophysiological evidence of neuromuscular transmission defect [10]. This study suggests that GAD antibodies may lead to a defect in nerve signal transmission, contributing to muscle and overall fatigue. A pilot study analyzed individuals with autoimmune encephalitis and SPS, both of which have GAD antibodies, who presented with hypersomnia. Hypersomnia was defined as a mean sleep latency of <8 minutes during multiple sleep latency tests (MSLT). They found that a higher number of patients experienced sleep attacks (three versus zero) and an excessive need for sleep (five versus zero), in comparison to controls [11]. The results of this study suggest that central hypersomnia is a pertinent characteristic in individuals with anti-GAD neurological disorders. This necessitates a need for further research on sleep disorders and their relationship to anti-GAD neurological disorders to improve patient management and quality of life.

The two main treatment options for individuals with SPS are GABAergic therapy or immunotherapy. First-line treatment is usually a benzodiazepine, due to its enhancement of GABA, leading to reduced nerve excitability and enhanced muscle relaxation. Other GABAergic medications such as baclofen, cyclobenzaprine, and Robaxin may also be used. The first choice of antispasmodics is baclofen in combination with diazepam [12]. The patient presented in this case was on baclofen and clonazepam, which are longer acting in comparison to diazepam. She was also receiving IVIG infusions every three weeks. IVIG is often used for the chronic maintenance of SPS. One study found that in patients with monthly maintenance IVIG, 24 of 36 patients (67%) had a clinically meaningful response over a median 3.3-year period. They exhibited improved gait, posture, and balance and decreased stiffness, spasms, and startle responses [13]. This further highlights the importance and benefit of long-term IVIG therapy.

## Conclusions

This case highlights the rarity of stiff person syndrome and the diagnostic challenges that come due to its highly variable presentation. SPS is heterogeneous in its presentation, progression, and etiology. Recognizing key diagnostic features of this disease can help physicians make timely diagnoses and improve patient outcomes. The early detection of SPS can greatly impact one’s prognosis and quality of life. The goal of this case study was to enhance the understanding of the complexity of SPS in hopes that clinicians would continue exploring various symptomatology, testing modalities, and treatments for these patients. As research continues, refining diagnostic modalities will prove to be critical for improving outcomes in patients with SPS.
